# Association between Increasing Social Capital and Decreasing Prevalence of Smoking at the Municipality Level: Repeated Cross-Sectional Study from the JAGES

**DOI:** 10.3390/ijerph19084472

**Published:** 2022-04-08

**Authors:** Hiroki Takeuchi, Kazushige Ide, Ryota Watanabe, Yasuhiro Miyaguni, Katsunori Kondo

**Affiliations:** 1 Graduate School of Medicine and Pharmaceutical Sciences, Chiba University, 1-8-1 Inohana, Chuo-ku, Chiba-shi 260-0856, Chiba, Japan; 2 Department of Social Preventive Medical Sciences, Center for Preventive Medical Sciences, Chiba University, 1-33 Yayoicho, Inage-ku, Chiba-shi 263-8522, Chiba, Japan; ide.k@chiba-u.jp (K.I.); ryota.watanabe.1222@gmail.com (R.W.); kkondo@chiba-u.jp (K.K.); 3 Department of Frailty Research, Center for Gerontology and Social Science, Research Institute, National Center for Geriatrics and Gerontology, 7-430 Morioka-cho, Obu City 474-8511, Aichi, Japan; 4 Faculty of Social Welfare, Nihon Fukushi University, 35-6 Okudaikai, Mihama-cho, Chita-gun 470-3295, Aichi, Japan; miyaguni@n-fukushi.ac.jp; 5 Department of Gerontological Evaluation, Center for Gerontology and Social Science, Research Institute, National Center for Geriatrics and Gerontology, 7-430 Morioka-cho, Obu City 474-8511, Aichi, Japan

**Keywords:** smoking, health promotion, older people, social participation, social support, age-friendly city, healthy aging

## Abstract

As smoking cessation is crucial for both individual and public health, this study aimed to elucidate the association between changes in social capital and in terms of smoking at the level of municipal units in Japan. Using repeated cross-sectional data from the Japan Gerontological Evaluation Study, we analyzed data from adults aged 65 years or older from 69 municipal units that participated in two survey waves. We received valid responses from 91,529 and 86,403 older people in 2013 and 2019, respectively, and aggregated all variables by municipal units. For the dependent variable, we used the units’ prevalence of smoking for both years. The independent variables were the percentages of social capital indicators, such as social participation, social cohesion, and reciprocity for each of the 69 municipal units. The mean prevalence of smoking increased from 9.7% in 2013 to 10.2% in 2019. Multiple regression analysis revealed that increases in the percentages of sports group participation, receiving emotional and instrumental social support, and reciprocity were significantly associated with decreased prevalence of smoking, after we adjusted for confounding variables. This study indicates that building social capital might be useful in promoting smoking cessation and that its indicators could be useful in monitoring efforts.

## 1. Introduction

Smoking is a major behavioral risk factor for disability-adjusted life years, and mitigating and, ultimately, eliminating this risk requires both individual-level and environmental approaches [1]. One of the World Health Organization’s (WHO) strategic goals for healthy aging [2] is creating what it terms age-friendly cities and communities (AFCs) that are comfortable for older people to live in [3,4]. Additionally, one of the action areas under the WHO’s Decade of Healthy Aging initiative is fostering the abilities of older people [5,6], which includes promoting health and building and maintaining physical and mental capacity throughout the life course. One major activity toward the aim of improving older persons’ health is smoking cessation [5,6].

In the AFC evaluation and monitoring framework [7], social participation is not only one of the eight core intervenable indicators, but also one of the components of social capital [8]. Social capital can be categorized as individual or collective; collective social capital is based on social cohesion in the environment [9]. At the lower level of social capital, it can be structural, such as social participation, or cognitive, such as trust, norms, support, and reciprocity [8].

Regarding the association between social capital and smoking cessation, the authors of a study on an 8-year intervention in adults reported that belonging to a group led to individual smoking cessation and to the maintenance of smoking cessation [10]. In a study of adolescents, trust and reciprocity within individuals’ in-groups showed some encouraging effects on individual smoking cessation [11,12]. Additionally, researchers found that living in an area where social participation, such as in sports, was prevalent made individuals more likely to quit smoking, even if they themselves did not participate in the social activities [13]. These findings suggest that measures to increase social capital at the community level can serve as smoking control measures as well. Nevertheless, as yet, there have been no examinations of the association between increased community-level social capital, a component of AFCs, and a decrease in the prevalence of community-level smoking. Thus, we conducted this study to examine whether social capital could serve as an indicator of an AFC by elucidating whether smoking is decreasing in municipalities that have richer social capital at the municipality level.

## 2. Materials and Methods

### 2.1. Data

We used repeated cross-sectional data from the Japan Gerontological Evaluation Study (JAGES) [14,15], an ongoing cohort study focusing on social and behavioral factors associated with health decline of older people. JAGES is a study on the social determinants of health and social capital. It is one of the few population-based gerontological repeat surveys in Japan that focuses on the environment. We collected our data from the 2013 and 2019 waves of the JAGES. For the survey, self-reported questionnaires were mailed to people aged 65 years or older (2013; *n* = 193,694 respondents in 81 municipal units, 2019; *n* = 367,640 respondents in 122 municipal units) who were not certified as receiving public long-term care insurance benefits. We received, respectively, 137,736 and 260,250 completed surveys, for response rates of 71.1% and 70.8%, respectively. Of these, we used data from 69 municipal units (2013; *n* = 100,139, 2019; 98,762) that participated at both time points. We excluded missing information on smoking (2013; *n* = 1298, 2019; 1380), activities in daily living (ADLs; 2013; *n* = 4188, 2019; 5263), and municipality (2013; *n* = 0, 2019; 601); we also excluded responses from individuals whose ADLs were not independent (2013: *n* = 3124; 2019: 5115). For both 2013 and 2019, no missing information on sex or age was observed in the data set used in this study. From the 69 municipal units from both waves, we were left with 91,529 respondents from 2013 (minimum: 205 per unit, maximum: 7150) and 86,403 from 2019 (minimum: 208, maximum: 5519) for the final analysis. The 69 municipal units analyzed in this study include wards (ordinance-designated cities) with functions similar to those of municipalities, in addition to municipalities based on living areas established by each prefecture.

Random sampling was used to administer the surveys in 61 of the municipal units we studied, and respondents aged 65–67 years were selected in 2013 for follow-up in 2019 to generate panel data. In the remaining eight municipal units, complete enumeration survey methods were conducted: all people aged 65 years or older who were not certified as requiring long-term care were surveyed in both 2013 and 2019. Informed consent from participants was given in writing; in 2013, consent was given while replying to a questionnaire, and in 2019, consent was given by checking a consent checkbox in accordance with changes in the ethical review policy in Japan. We obtained ethical approval for the study from the Nihon Fukushi University Ethics Committee (application number: 13-14), National Center for Geriatrics and Gerontology (application number: 992), Chiba University (application number: 2493).

### 2.2. Dependent Variables

For the dependent variables of this municipality-level study, we subtracted the self-reported prevalence of smoking in 2013 from that in 2019 on the basis of the response “I smoke” to the question “Do you smoke?” In 2013, we defined smokers as respondents who selected “I smoke,” from among the answers “I smoke,” “I quit,” or “I do not smoke” in response to “Do you smoke?” [16,17]. In 2019, we defined smokers as respondents who selected “I smoke almost every day” or “I smoke occasionally” from among the answers “I smoke almost every day,” “I smoke occasionally,” “I quit within 5 years and don’t smoke now,” “I quit more than 5 years ago and don’t smoke now,” and “I never smoked” in response to “Do you smoke?” [16,17].

### 2.3. Independent Variables

For the independent variable, we measured social capital indicators in 2013 and 2019 and subtracted the 2013 percentages from those for 2019 for each of the 69 municipal units. For this study, we defined social capital, a concept that has been used in epidemiological studies [18], as “resources that are accessed by individuals as a result of their membership of a network or a group” [8].

We based our selection of social capital indicators on Saito et al. [19]. For social participation, we assessed the frequency of participation in a sports group, a volunteer group, a hobby activity or a study, education group, or in teaching a skill; we asked, “How often do you participate in the following associations/groups?” and added the response option of at least once a month. For social network, we assessed the frequency of contact with friends and acquaintances; we asked, “How often do you meet with friends and acquaintances?” and added the response option of at least once a month. For social support, we measured respondents’ percentage of receiving and providing emotional and instrumental social support using two questions for each. For emotional social support, the questions were “Do you have someone who listens to your concerns and complaints?” and “On the contrary, do you have someone whose concerns and complaints you listen to?” For instrumental social support, we used the questions “Do you have someone who will take care of you when you are sick and in bed for a few days?” and “On the contrary, do you have someone you take care of when she or he is sick in bed?” All the social support responses were binary (yes or no). Using these variables, we calculated the Social Capital Index [19]. In addition, Saito et al. measured social capital on a regional basis using civic participation (five questions), social cohesion (three questions), and reciprocity (three questions). Civic participation refers to residents’ participation in community organizations and activities (in a sports group, a volunteer group, a hobby or a study, an education group, or teaching a skill); social cohesion pertains to the cognitive aspects of interpersonal trust, which comprises community trust, community contribution, and community attachment; reciprocity represents community social support (receiving and providing emotional social support and receiving instrumental social support). These factors are calculated as follows: Civic participation = %Volunteer group × 0.6 + %Sports group × 0.8 + %Hobby activity × 0.9 + %Study or education group × 0.7 + %Skill teaching × 0.5, Social cohesion = %community trust × 0.9 + %community contribution × 0.8 + %community attachment × 0.7, Reciprocity = %Receiving emotional social support × 0.8 +%Providing emotional social support × 0.7 + %Receiving instrumental social support × 0.6 [19].

### 2.4. Covariates

The adjusted variables related to socioeconomic status in both waves. Specifically, in both waves, we measured the proportions of respondents who had fewer than 10 years of education, who were employed, who were living alone, and who had an equivalized household income of less than 2 million yen [17,20,21,22]. We then used the changes in these variables in our analyses.

### 2.5. Statistical Analysis

A simple comparison of values aggregated at the municipal level depends on the differences in the age structure of each municipality [23,24,25]. In general, municipalities with larger populations of older people tend to have poorer health outcome results, while municipalities with smaller populations of older people tend to have better health outcome results. Therefore, in order to compare various indicators across municipalities with different age structures, the age standardization method used by the Ministry of Health, Labor and Welfare was applied in this study, and all variables were adjusted for age [26]. This age-adjusted variable facilitated the accurate comparison of regions without focusing on differences in the age structure within groups. The formula is as follows [26]: Regional age adjustment index rate = {(regional coarse index ※ rate by age of 5 years × the population of the age group of reference population) × the sum total of each age class of the indicator}/total number of reference population. ※ The coarse index rate is obtained by dividing each indicator by the population [26]. Second, in the multivariate analysis, we used multiple linear regression models to examine the associations between changes in social capital indicators as well as in prevalence of smoking. In the association between changes in social capital indicators and changes in prevalence of smoking, social capital indicator items were input separately, and partial regression coefficient (B) and 95% confidence interval (CI) were calculated. In the crude model, changes in indicators and prevalence of smoking were analyzed with separate inputs, and in Model 1, we added the covariates for the changes in the adjusted socioeconomic variables. We conducted all analyses using StataMP16.1 (StataCorp, College Station, TX, USA)

## 3. Results

Table 1 presents the descriptive statistics for each variable for 2013 and 2019. The mean prevalence of smoking increased by 0.5%, from 9.7% in 2013 to 10.2% in 2019, and the prevalence of smoking changes ranged from −4.1% to 3.8%, a difference of 7.9 percentage points. The change in mean in social capital from 2013 to 2019 was as follows: sports group (3.5%), volunteer group (1.8%), hobby activity (1.0%), study or education group (0.0%), skill teaching (0.3%), frequency of contact with friends (−0.3%), receiving emotional social support (0.3%), providing emotional social support (1.4%), receiving instrumental social support (0.2%), providing instrumental social support (1.9%), civic participation (5.0 points), social cohesion (5.0 points), and reciprocity (3.5 points).

Table 2 shows the changes in the prevalence of smoking associated with changes in social capital, analyzed using multiple linear regression models. The crude model showed significant associations between decreased prevalence of smoking and 6 of the 13 social capital indicators: sports group participation at least once per month (B, −0.17), receiving emotional social support (−0.42), providing emotional social support (−0.25), receiving instrumental social support (−0.32), social cohesion (−0.07), and reciprocity (−0.21). In Model 1, we entered changes in the percentage of education ≤ 10 years, percentage of employed, percentage of living alone, and percentage of equivalent income ≤ 2,000,000 JPY, and the results showed significant associations (*p* < 0.05) between decreased prevalence of smoking and 4 of the 13 social capital indicators: sports group participation at least once per month (−0.17), receiving emotional social support (−0.45), receiving instrumental social support (−0.28), and reciprocity (−0.23).

## 4. Discussion

No previous studies have examined the association between social capital and smoking at the municipality level, which is a component of the WHO’s age-friendly communities, or focused on changes in social capital and the prevalence of smoking among older people. This is the first study to use repeated cross-sectional data to show that community prevalence of smoking decreased in municipalities where municipality-level social capital increased, which in this case, was among Japanese adults aged 65 years or older from 69 municipal units that participated in both the 2013 and 2019 waves of the JAGES. By analyzing the change in certain social capital indicators between two time points, we determined that improvements in municipality-level social capital—such as sports participation, receiving emotional and instrumental social support, and experiencing reciprocity in the municipality—were associated with decreased municipality prevalence of smoking, even after accounting for the effects of unchanging community characteristics. This indicated that social capital indicators might be useful as monitoring indicators in the effort to promote smoking cessation through age-friendly community measures.

Regarding the prevalence of smoking in Japan, it has decreased on a decadal basis but not necessarily on a yearly basis; national surveys in 2013 and 2019 show an increase in smoking prevalence in 2019 over 2013 [27]. The results of our survey suggest that this trend has been captured. In addition, the prevalence of heat-not-burn tobacco electronic cigarettes is increasing in Japan [28], which might be captured by this trend. Furthermore, the results of our study suggest that changes in the prevalence of smoking range from increases to decreases, with the prevalence of smoking decreasing in municipalities where social capital increased.

Regarding the increased mean percentage of social participation, one of the reasons behind this increase is the prevention of long-term care and smoking cessation through community development. In Japan, the limitations of the high-risk approach strategy in the prevention of long-term care and smoking cessation and other strategies have become apparent, and the population approach strategy has been promoted since 2015 [29]. Specifically, community development expands opportunities for resident-oriented social participation and includes sports groups and learning and culture, conducted by departments other than the local government [30]. Consequently, it has been shown that, in recent years, there has been an increase in the social participation of older people [31]. The results of our study support these efforts and results.

Regarding the association between social capital and smoking cessation, in a cross-sectional study of adults that focused on the association between social capital and smoking at the individual level, social participation, network, and trust were positively associated with smoking cessation [32]; in a cross-sectional study of adolescents, emotional and practical support had the same positive associations [33]. In a cohort study, conducted using data from the British Household Panel Survey, social participation led to smoking cessation [12], and in other cohort studies of adults, support from family or a spouse also did so [34]. In studies on the association between social capital and individual smoking at the community level, trust and reciprocity in an individual’s in-group increased individual smoking cessation [11,12], and living in a community where sports and other activities are popular led to smoking cessation, even among individuals who did not participate in such activities [13]. The results of this study showed that participation in community-level sport groups at least once per month, receiving emotional and instrumental social support, and experiencing reciprocity were associated with decreased community prevalence of smoking, thus, supporting the findings of previous studies. Additionally, although the associations were not significant, municipality-level prevalence of smoking decreased the percentages in municipality-level volunteering, hobbies, and study or education groups, providing emotional and instrumental social support and experienced social cohesion. In addition, previous studies have shown that social capital, such as social cohesion and reciprocity [11,16,32,33,34], is associated with a lower prevalence of smoking. The results of our study support these previous studies.

The known mechanisms of municipality-level social capital associated with health outcomes have been described as (1) social contagion, (2) informal social control, and (3) collective efficacy [8]. Social contagion describes the concept that habits and behaviors spread through dense social networks [8]; Christakis et al. found that smoking cessation was “influenced by three-dimensional connections” and that networks rich in social capital indicators, such as cohesion and reciprocity, diffused the behavior [35]. Informal social control refers to the ability of community members to maintain order in the community and has social capital aspects, such as group and community trust and reciprocity [8]. Collective efficacy raises the concept of self-efficacy to the group level to indicate how well group members can work together in solidarity [8].

Individual-level cohort studies have shown that social participation increases physical activity [36], access to health information [37], and health awareness [38,39], which combine to promote smoking cessation [40,41], and it is possible that these factors observed in individuals may spread to the community through social contagion, informal social control, and collective efficacy. Findings from one community-level study also indicated that improvements in community depression led to community smoking cessation in a cross-sectional study [42], and other researchers found that the prevalence of depressive symptoms decreased in municipalities with increased social participation and reciprocity in repeated cross-sectional study and a longitudinal study [25,43]. These results indicate that using the AFC construct to improve social capital is useful as a measure to promote smoking cessation, by increasing four social capital indicators: sports group participation, receiving emotional and instrumental social support, and experiencing reciprocity.

This study has four strengths, and the first is that, although ours was not a representative sample of Japanese adults overall, it was a representative sample of 69 municipal units with various characteristics in 11 prefectures across Japan. The second strength is that the results of the analysis are more revealing than cross-sectional data from a single time point because we captured changes between two time points. Furthermore, the use of change between two time points allows for analysis that eliminates the influence of unchanged regional characteristics [44]. The third strength is that we consider the association with socioeconomic status and living alone, which have been considered to influence smoking. Previous studies have shown that the prevalence of smoking is higher among those with low socioeconomic status [17,21,22] and those living alone [20]. In this study, we show that even when socioeconomic status and living alone are considered, we found that increasing municipality-level social capital was associated with decreased municipality-level prevalence of smoking. Finally, to the best of our knowledge, this is the first study to elucidate the association between changes in municipality-level ecological social capital and prevalence of smoking. Previous longitudinal studies at the individual level have highlighted that social capital is associated with lower prevalence of smoking [10,16,34,35], suggesting that it may also be associated with lower prevalence of smoking at the community level. We identified the association between changes in ecological social capital at the municipal level and prevalence of smoking, and we were the first to examine the usefulness of social capital indicators in promoting smoking cessation through the lens of creating AFCs.

However, this study also has three limitations. The first limitation is that the analysis was conducted in 69 municipal units. Therefore, social capital indicators could not be analyzed simultaneously, independence could not be verified, and many factors could not be adequately adjusted. The second limitation is that because this was a repeated cross-sectional study, we could not rule out reverse causality; that is, that health-related social capital in the municipality increased because municipality smoking decreased. The results of this study capture a decrease in the prevalence of smoking at the municipal level in areas where social capital at the municipal level increased. Therefore, no mention of the temporal context could be made. On the other hand, a number of previous longitudinal studies found that social capital is associated with lower incidence of smoking, and there is a large literature suggesting a temporal before/after relationship [10,16,34,35]. Finally, it is uncertain that the findings of this study are applicable outside of Japan. However, findings that indicate the association between social capital and a lower prevalence of smoking in individual-level studies conducted outside Japan have also been accumulated [10,16,34,35].

## 5. Conclusions

With our study, using repeated cross-sectional data from 2013 and 2019, we are the first to examine the promotion of smoking cessation through age-friendly city measures and the usefulness of social capital indicators in monitoring smoking cessation. We found that increasing municipality-level social capital, such as the percentage of sport groups meeting at least once per month, receiving emotional and instrumental social support, and experiencing reciprocity, were associated with decreased municipality-level prevalence of smoking. This suggests that social capital indicators, such as sport groups meeting at least once per month, receiving emotional and instrumental social support, and experiencing reciprocity, might be useful for promoting smoking cessation as a monitoring indicator through age-friendly community measures.

## Figures and Tables

**Table 1:** Age-adjusted descriptive statistics factors (Municipal units = 69).

Variable	2013				2019				Difference (2019–2013)			
	Min	Max	Mean	SD	Min	Max	Mean	SD	Min	Max	Mean	SD
% Smoking ^a^	5.8	14.9	9.7	1.6	6.6	16.8	10.2	1.9	−4.1	3.8	0.5	1.7
%Sports group ^a,b^ (≥1 per month)	13.6	38.7	25.6	4.9	17.1	37.0	29.1	4.7	−4.1	11.2	3.5	2.9
%Volunteer group ^a,b^ (≥1 per month)	8.5	18.7	12.7	2.2	9.8	20.0	14.5	2.2	−3.4	6.9	1.8	2.1
%Hobby activity ^a,b^ (≥1 per month)	23.1	44.4	34.5	4.5	22.1	43.8	35.5	4.9	−7.6	8.4	1.0	3.2
%Study or education group ^a,b^ (≥1 per month)	4.7	16.7	10.4	2.4	5.3	17.3	10.4	2.5	−4.2	4.8	0.0	2.0
%skill teaching ^a,b^ (≥1 per month)	3.4	10.5	6.4	1.4	2.9	11.6	6.7	1.4	−3.6	4.3	0.3	1.5
%Frequency of contact with friends ^a,b^ (≥1 per month)	61.3	84.4	69.8	4.5	62.8	81.3	69.6	3.8	−7.4	6.9	−0.3	2.8
%Receiving emotional social support ^a^	89.6	97.7	94.2	1.4	90.7	97.5	94.6	1.4	−3.3	4.4	0.3	1.5
%Providing emotional social support ^a^	87.5	94.8	92.0	1.6	89.8	96.4	93.5	1.3	−3.0	5.8	1.4	1.7
%Receiving instrumental social support ^a^	87.4	98.3	94.7	2.2	89.1	97.6	94.5	1.8	−3.6	4.9	−0.2	1.7
%Providing instrumental social support ^a^	73.1	85.4	78.9	2.6	67.9	82.9	77.0	3.4	−8.3	5.2	−1.9	2.9
Civic participation ^a^	44.0	93.1	69.6	9.8	49.3	93.9	74.6	10.5	−12.0	22.4	5.0	6.2
Social cohesion ^a^	134.8	190.0	160.3	10.6	137.5	187.0	163.8	10.3	−12.3	18.7	3.5	5.9
Reciprocity ^a^	186.3	202.3	196.6	3.0	191.2	202.9	197.8	2.7	−4.7	9.5	1.1	3.0
%Education ≤ 10 years ^a^	14.0	64.5	35.3	12.7	7.7	46.2	22.7	8.8	−31.0	−2.3	−12.7	5.7
%Employed ^a^	13.5	32.0	21.5	3.3	18.4	37.2	28.1	3.6	0.0	12.6	6.6	2.5
%Living alone ^a^	7.9	31.3	16.8	5.5	8.0	35.6	17.1	5.2	−7.7	6.1	0.3	2.5
%Equivalent income ≤ 2,000,000 JPY ^a^	28.8	68.8	48.0	9.2	24.8	69.8	43.7	8.3	−15.1	7.9	−4.2	4.9

Min: Minimum, Max: Maximum, SD: Standard deviation. ^a^ All factors were adjusted for age using the age standardization method. ^b^ Percentage of respondents that participated in the group.

**Table 2:** Multiple linear regression of changes in health-related social capital indicators with changes in smoking (*n* = 69 municipal units).

Variables		Crude				Model 1 ^c^		
	B	95% CI		*p*	B	95% CI		*p*
%Change in ^a^ sports group (≥1 per month)	−0.17	−0.31	−0.04	0.012	−0.17	−0.31	−0.03	0.019
%Change in ^a^ Volunteer group (≥1 per month)	−0.11	−0.30	0.08	0.256	−0.12	−0.31	0.08	0.239
%Change in ^a^ Hobby activity (≥1 per month)	−0.09	−0.21	0.03	0.157	−0.07	−0.20	0.06	0.266
%Change in ^a^ study or education group (≥1 per month)	−0.03	−0.24	0.17	0.735	−0.02	−0.23	0.20	0.878
%Change in ^a^ skill teaching (≥1 per month)	0.03	−0.24	0.30	0.840	0.04	−0.23	0.31	0.769
%Change in ^a^ frequency of contact with friends (≥1 per month)	−0.01	−0.15	0.14	0.922	0.01	−0.14	0.17	0.863
%Change in ^a^ receiving emotional social support	−0.42	−0.67	−0.18	0.001	−0.45	−0.74	−0.17	0.002
%Change in ^a^ providing emotional social support	−0.25	−0.47	−0.02	0.033	−0.24	−0.50	0.03	0.078
%Change in ^a^ receiving instrumental social support	−0.32	−0.55	−0.09	0.006	−0.28	−0.55	−0.01	0.044
%Change in ^a^ providing instrumental social support	−0.06	−0.12	0.00	0.061	−0.06	−0.12	0.01	0.099
Change in ^b^ civic participation	−0.08	−0.22	0.06	0.242	−0.04	−0.21	0.12	0.598
Change in ^b^ social cohesion	−0.07	−0.13	0.00	0.049	−0.06	−0.14	0.02	0.120
Change in ^b^ reciprocity	−0.21	−0.34	−0.09	0.001	−0.23	−0.38	−0.07	0.005

All factors were adjusted for age using the age standardization method. ^a^ Percentage in 2019 subtracted from percentage in 2013. ^b^ Scores from 2019 subtracted from scores from 2013. ^c^ Model 1 was adjusted for change in living alone, change in educational attainment ≤10 years, change in equivalent income ≤ 2,000,000 JPY, and change in employed rate.

## Data Availability

The data were obtained from the JAGES. Inquiries regarding the data should be directed to the Data Management Committee (e-mail: dataadmin.ml@jages.net). All JAGES data sets contain confidential information about subjects, and ethical or legal restrictions are imposed on their release. These restrictions have been set under the guidance of the local authorities who contributed to the research. Researchers may use the data by submitting a research plan (JAGES Data Use) and obtaining approval for data use.
